# Transportation and Fuels: Ethanol Boosts Gas Engines

**DOI:** 10.1289/ehp.115-a446b

**Published:** 2007-09

**Authors:** David C. Holzman

The gasoline internal combustion engine has more than 100 years of intense development behind it. But now three researchers from the Massachusetts Institute of Technology (MIT) have modified it in a way that elevates efficiency by a remarkable 25%, an advance that could greatly mitigate greenhouse gas emissions and offers compelling advantages over hybrids and diesels. “This has real potential,” says David Cole, chairman of the Center for Automotive Research, a nonprofit organization of the University of Michigan.

The design logic is simple. One can alter an engine to create greater compression of the fuel/air mixture within each cylinder, raising thermodynamic efficiency. One can also add a turbocharger, which force-feeds more fuel/air mixture into the cylinders. This makes it possible to get more power out of an engine, or to downsize an engine without losing power, making it still more efficient.

The problem: boosting compression also boosts temperature, and too much heat can ignite the fuel/air mixture prematurely, causing potentially damaging engine “knock.” But Daniel Cohn and Leslie Bromberg of MIT’s Plasma Science and Fusion Center, and John Heywood of MIT’s Sloan Automotive Lab figured out that a little squirt of ethanol into the cylinder from a separate tank could cool it in the same way that rubbing alcohol cools the skin—by vaporizing, then absorbing excess heat. The researchers have formed a company, Ethanol Boosting Systems (EBS), and have drawn several prominent figures on board, including Neil Ressler, former chief technology officer of Ford Motor Company.

**Table:** How Alternative Engines Stack Up (compared with conventional gasoline engines, except as noted)\

	**Clean Diesel**	**Electric Hybrid**	**Ethanol**
**Cost**	$3,000–3,500^a^	$3,500–5,000 + possible battery replacement cost^a^	$1,000–1,500^a^
**Efficiency Gain**	20–30% more efficient^a^	30–40% more efficient^a^	20–30% more efficient^a^
**Emissions**	25% lower CO_2_ emissions^b^	Up to 50% lower CO_2_ emissions^c^	NO_x_ and PM reduction, compared with clean diesel^a^
**Technological Advantages**	Better engine performance^d^	Better engine performance^e^	Reduced engine weight, more space in engine compartment, compared with electric hybrid^a^
	Less complex and easier to install than EH engine^b^	Larger battery means more safety and luxury electronic systems can be added on^e^	Higher torque and horsepower, compared with clean diesel^a^

a http://www.ethanolboost.com/Technology.htm

b http://www.signonsandiego.com/uniontrib/20060207/news_1n7diesel.html

c http://www.ama.ab.ca/cps/rde/xchg/ama/web/advocacy_safety_envt_hybrid.htm

d http://www.discoveralternatives.org/Alternative_Fuel_Autos_Technology.php

e http://www.alliancebernstein.com/CmsObjectABD/PDF/Research_WhitePaper/R37755_Hybrid.pdf

According to *Calculations of Knock Suppression in Highly Turbocharged Gasoline/Ethanol Engines Using Direct Ethanol Injection*, a 2006 MIT report, bench engine tests by Ford show that the knock limit can be vastly alleviated, and unpublished results indicate that a 25% increase in efficiency should therefore be attainable. That would reduce carbon dioxide emissions by about 20%, says Cohn. The engine’s alcohol consumption would be minimal, because the extra cooling is unnecessary under light loads, such as steady driving at low to moderate speeds.

Although not quite as efficient as the best full hybrid systems, the EBS is far simpler, because it needs no electric motor, extra batteries, or complex software. Cohn says those factors would shave $2,000–4,500 off the cost relative to a full hybrid. The EBS and full hybrid systems would have similar emissions profiles.

An EBS engine would also be a couple of thousand dollars cheaper than a diesel engine. The two engines would produce roughly the same amount of greenhouse gas emissions, but the EBS would otherwise be cleaner, emitting fewer nitrogen oxides (NO_x_) than the diesel engine, and less particulate matter. Many U.S. cities have nonattainment zones for NO_x_, which contributes to ground-level ozone and can damage lung tissue and vegetation.

In a column in the July 2007 issue of *Car and Driver*, editor-in-chief and engineer Csaba Csere praises the EBS technology and says that if some seemingly manageable problems are solved—for example, maintaining fuel economy under real-world conditions of elevated temperatures and substandard fuel—EBS engines could be powering cars early in the next decade.

